# Protective Effect of Renin-Angiotensin System Inhibitors on Parkinson’s Disease: A Nationwide Cohort Study

**DOI:** 10.3389/fphar.2022.837890

**Published:** 2022-03-03

**Authors:** Youngkwon Jo, Seungyeon Kim, Byoung Seok Ye, Euni Lee, Yun Mi Yu

**Affiliations:** ^1^ Department of Pharmacy and Yonsei Institute of Pharmaceutical Sciences, College of Pharmacy, Yonsei University, Incheon, South Korea; ^2^ College of Pharmacy and Research Institute of Pharmaceutical Sciences, Seoul National University, Seoul, South Korea; ^3^ Transdisciplinary Department of Medicine and Advanced Technology, Seoul National University Hospital, Seoul, South Korea; ^4^ Department of Neurology, Yonsei University College of Medicine, Seoul, South Korea; ^5^ Department of Pharmaceutical Medicine and Regulatory Sciences, Colleges of Medicine and Pharmacy, Yonsei University, Incheon, South Korea

**Keywords:** Parkinson’s disease, renin-angiotensin system, myocardial ischemia, neuroprotective agents, pharmacoepidemiology

## Abstract

**Background:** Renin-angiotensin system (RAS) inhibitors have been suggested as protective agents in Parkinson’s disease (PD). However, epidemiological evidence on the association between RAS inhibitors and the development of PD is inconsistent.

**Objectives:** To investigate the effect of RAS inhibitors on PD risk in patients with ischemic heart disease (IHD) by type and cumulative duration of RAS inhibitors and their degree of blood-brain barrier (BBB) penetration ability.

**Methods:** This was a propensity score-matched retrospective cohort study using 2008–2019 healthcare claims data from the Korean Health Insurance Review and Assessment database. The association between RAS inhibitor use and PD in patients with IHD was evaluated using multivariate Cox proportional hazard regression analysis. The risks are presented as adjusted hazard ratios (aHRs) and 95% confidence intervals (CIs).

**Results:** Over a 10-year follow-up, 1,086 of 62,228 IHD patients developed PD. The Cox regression model showed that the use of RAS inhibitors was significantly associated with a lower risk of PD (aHR = 0.75; 95% CI 0.66–0.85) than the non-use of RAS inhibitors. Specifically, this reduced risk of PD only remained with the use of BBB-crossing angiotensin II receptor blockers (ARBs) (aHR = 0.62; 95% CI = 0.53–0.74), and this association was more definite with an increasing cumulative duration. A significantly reduced risk of PD was not observed with the use of BBB-crossing angiotensin-converting enzyme inhibitors.

**Conclusions:** The use of ARBs with BBB-penetrating properties and a high cumulative duration significantly reduces the risk of PD in IHD patients. This protective effect could provide insight into disease-modifying drug candidates for PD.

## Introduction

Parkinson’s disease (PD) is the second most common neurodegenerative disease, and the worldwide prevalence was reported as 1% in people aged ≥60 years (Tysnes and Storstein, 2017). PD is pathogenetically characterized by a gradual loss of dopaminergic neurons from the substantia nigra pars to the striatum, resulting in reduction of dopamine to the striatum (Samii et al., 2004; Connolly and Lang, 2014). Despite the effective symptomatic treatment for PD by restoring the dopamine level or activating the receptors, multiple trials to discover disease-modifying therapy have failed in the clinical phase (Kalia et al., 2015). As PD has traditionally been regarded irreversible, there is a growing number of unmet therapeutic need to delay disease progression or prevent premature mortality. Research on novel therapies to slow the neurodegenerative progression or to prevent PD in the early stages has been conducted (Kalia et al., 2015). Specifically, a number of studies have suggested various medications including statins (Kumar et al., 2012; Kang et al., 2017), phosphodiesterase inhibitors (Nthenge-Ngumbau and Mohanakumar, 2018; Erro et al., 2021), and renin-angiotensin system (RAS) inhibitors (Ana Flavia et al., 2017; Victor Teatini Ribeiro, 2020) as drug repurposing candidates with demonstrated neuroprotective effects.

The RAS is a circulating hormonal system responsible for the regulation of systemic blood pressure, electrolyte homeostasis, and volume status, which exerts its function via two main receptors: angiotensin (Ang) II type 1 (AT_1_) and type 2 (AT_2_) (Oro et al., 2007). These two receptors are present in various tissues, including the brain (Rivas-Santisteban et al., 2020). Recent evidence from animal studies showed that inhibition of brain RAS is associated with decreased dopaminergic neuronal loss and reduced oxidative stress in PD via the via alleviation of mitochondrial function (Perez-Lloret et al., 2017; Ray et al., 2021). Brain RAS blockade may play a protective role in neuroinflammation and accumulation of phosphorylated alpha-synuclein. This is accomplished by blocking the Ang II-related pro-inflammatory oxidative effects and reducing the production of reactive oxygen species associated with AT_1_ receptor overexpression in the substantia nigra (Wright and Harding, 2012; Perez-Lloret et al., 2017; Ray et al., 2021).

Although the role of RAS inhibition in preventing PD is well documented in animal and *in vitro/in vivo* studies, only a few human studies have focused on the association between RAS inhibitors and PD, and the results have been inconsistent. While some clinical studies have found evidence of the neuroprotective effect of RAS inhibitors (Y.-C. Lee et al., 2014; Reardon et al., 2000), others have shown no association between RAS inhibitor use and PD (Becker et al., 2008; Ritz et al., 2010; Warda et al., 2019). Moreover, considering the potential influence of the duration of RAS inhibitors and differences in central nervous system penetration among these agents (Wu et al., 2016; Ouk et al., 2021), additional clinical evidence based on a valid study design and taking into account these factors is required to support the effect of RAS inhibitors on the incidence of PD.

Herein, we aimed to investigate the effect of RAS inhibitors on PD occurrence using a longitudinal national claims database in Korea. Specifically, we analyzed the cumulative duration-response relationship and compared the effect on PD based on RAS inhibitor type and blood-brain barrier (BBB) penetration.

## Materials and Methods

### Study Design and Data Source

This retrospective cohort study was conducted using national claims data from the Health Insurance Review and Assessment Service (HIRA) database between 1 January 2008 and 31 December 2019. The Republic of Korea has a single-payer health insurance system that provides compulsory universal health insurance that covers the entire population of Korea (J. A. Kim et al., 2017). Healthcare providers must submit medical claims to the HIRA to receive an evaluation of the appropriateness of the reimbursement. The HIRA generates a research-ready database containing sociodemographic characteristics and clinical information. The database includes diagnoses, procedures, and prescription records from submitted claims after anonymizing all participants according to strict confidentiality guidelines from the medical care claims of all patients.

This study was approved by the Institutional Review Board (IRB) of Yonsei University (IRB number: 7001988-202004-HR-846-01E). The requirement for informed consent was waived because the data were publicly available and de-identified.

### Study Population

To evaluate the effect of RAS inhibitors on the development of PD in patients with ischemic heart disease (IHD), we enrolled patients aged ≥60 years who were diagnosed with angina or myocardial infarction during the identification period (1 January 2009, to 30 June 2009). Patients diagnosed with angina or myocardial infarction were identified based on the International Classification of Diseases 10^th^ Revision (ICD-10) diagnostic codes I20.0-I22.0. We designated a specific time point (1 July 2009) as the index date for all the study participants. All study participants were followed-up on the index date.

To identify new users of RAS inhibitors during the identification period, patients who had prescription records of RAS inhibitors prior to the identification period or who had been prescribed their first RAS inhibitors after the identification period were excluded. Patients who had died, had last claims records, or were previously diagnosed with parkinsonism (G20-G26), including PD before the index date, were also excluded. Given that PD may have a prodromal period (Postuma and Berg, 2016), a 1-year lag time was included to minimize the reverse causality of PD. The new users of RAS inhibitors during the identification period were classified into user groups. Patients who had never been prescribed RAS inhibitors during the study period were classified into a non-user group.

### Exposure Assessment and Data Collection

The RAS inhibitors used in this study included angiotensin-converting enzyme inhibitors (ACEIs) and AT_1_ receptor blockers (ARBs), based on the Anatomic Therapeutic Chemical classification system of drugs by the World Health Organization (WHO) Collaborating Centre for Drug Statistic Methodology (World Health Organization, 2021). A detailed list of the RAS inhibitors is provided in Supplementary Table S1. To evaluate the cumulative effect of RAS inhibitors on the incidence of PD, we accumulated the doses and duration of RAS inhibitors during the follow-up period by collecting prescription information, including dosage, frequency, and days of supply.

The accumulated doses were calculated by multiplying the dosage, frequency, and number of supply days. These values were converted into the cumulative defined daily dose (cDDD) developed by the WHO (Methodology, 2021). The cumulative duration of RAS inhibitor use was calculated as the sum of the prescription days. The daily equivalent dosage, defined as the ratio of the cumulative dose to the cumulative exposure period, was calculated. To compare the preventive effect on PD, the patients were categorized into four groups according to their prescriptions: (1) use of non-BBB-crossing ACEIs, (2) use of BBB-crossing ACEIs, (3) use of non-BBB-crossing ARBs, and (4) use of BBB-crossing ARBs (Supplementary Table S1) (Alzahrani et al., 2020; C. K. ; Kim et al., 2015; Oka et al., 1988; Sink et al., 2009; Takai et al., 2004; Wharton et al., 2012; Yagi et al., 2013). These groups were based on the degree of BBB penetration and the type of RAS inhibitor prescribed.

Data on sociodemographic characteristics, including the patient’s age, sex, and insurance type (i.e., health insurance or medical aid), were collected during the identification period. Potential confounding factors affecting the development of PD, including comorbid diseases and concurrent medications, were recorded until the date of outcome occurrence or the end of the study period. These confounding factors were confirmed when patients had continuous claim records on diagnosis and prescription at least once per year until the last follow-up (J. Lee et al., 2018).

Comorbid diseases known as potential risk or protective factors for PD included hypertension, dyslipidemia, diabetes mellitus, end-stage renal disease, stroke, brain injury, dementia, chronic obstructive pulmonary disease (COPD), depression, gout, osteoarthritis, osteoporosis, and severe liver disease (Hu et al., 2020; Jafari et al., 2013; C. H. ; Li et al., 2015; Rugbjerg et al., 2010; Torsney et al., 2014; Tryc et al., 2013; Vale et al., 2018; Wang et al., 2018). Concurrent medications known to be potential risk or protective factors for PD include antipsychotics, antiemetics, calcium channel antagonists, antiepileptics, trimetazidine, dopamine depleters, mood stabilizers, beta-blockers, dihydropyridine calcium channel blockers, and statins (Ascherio and Schwarzschild, 2016; Jeong et al., 2019; S. ; Kim et al., 2020; López-Sendón et al., 2013; Mullapudi et al., 2016; Shin and Chung, 2012). The ICD-10 codes for comorbid diseases and details of the concurrent medications are listed in Supplementary Tables S2, 3.

### Outcome Measures

The primary outcome measure was new-onset Parkinson’s disease during the follow-up period. New-onset PD was defined as an ICD-10 diagnostic code for PD (G20), accompanied by the PD registration code and prescription records for any PD treatment drug. These include L-dopa, carbidopa/benserazide, pramipexole, ropinirole, bromocriptine, selegiline, rasagiline, amantadine, entacapone, trihexyphenidyl, and benztropine. In Korea, the government established a registry called the Rare and Intractable Diseases Program, which registered patients with neurologist-confirmed PD (Park et al., 2019). To enhance the validity of PD diagnosis in this study, we used the PD registration code (V124) for the outcome definition. The date of study outcome was defined as the date of the first appearance of the PD diagnostic code for G20.

### Statistical Analysis

To adjust for selection bias, we used a propensity score (PS)-matched design. PS was estimated using multivariate logistic regression. The predictors of PS included sex, age, insurance type, follow-up duration, comorbid diseases, and concurrent medications. Patients who did and did not use RAS inhibitors were matched in a 1:1 ratio without replacement, using the greedy method. This was within a caliper that was 0.2 times the standard deviation of the logit PS. After PS matching, the balance diagnostics between RAS inhibitor users and non-users was assessed by evaluating the standardized difference between them. When the standardized difference was >0.1, it was regarded as a sign of imbalance (Austin, 2009). Furthermore, the similar distribution of PS between RAS inhibitor users and non-users after matching is confirmed graphically in Supplementary Figure S1.

Descriptive statistics were used to summarize the characteristics of the matched population. Between-group comparisons of the distribution of sociodemographic data, comorbid diseases, and concurrent medications between RAS inhibitor users and non-users were performed using Pearson’s chi-squared test and Student’s *t*-test. The incidence rate of PD per 1,000 person-years was calculated by dividing the number of outcome events by the total number of person-years at risk and multiplying the result by 1,000. The person-years at risk were accumulated for each patient from the index date to the outcome date, death, or 31 December 2019, whichever occurred first.

Multivariable Cox proportional hazard regression analyses adjusted for demographic characteristics, comorbidities, and concurrent medications were used to estimate the hazard ratio (HR) and 95% confidence interval (CI) for the risk of PD in relation to RAS inhibitor use. The proportional hazard assumption was confirmed by a statistical test of the correlation between Schoenfeld residuals and survival time, using a cumulative log(minus) log curve (Supplementary Figure S2). To investigate the cumulative dose-response and duration-response relationships, the risk of PD was investigated according to the cumulative dose, total duration, and daily equivalent dosage of RAS inhibitors, using a multivariate Cox regression model. Additionally, the adjusted hazard ratios (aHRs) of PD were estimated according to the type of RAS inhibitor (i.e., ACEIs and ARBs) used and the degree of BBB penetration. Subgroup analyses were performed to evaluate the effect of RAS inhibitors on PD risk according to patient sex and age. Age was divided into two groups based on the mean age of the study population.

Sensitivity analyses were conducted using three steps. First, the index date from 1 July 2009 was shifted to 1 July 2010 and 2011. As the identification period changed accordingly, drug exposure, outcomes, and confounding factors were re-assessed. Second, the lag time was extended for 3 and 5 years, resulting in a decrease in the follow-up period and the number of study participants. As the lag time was extended, captured PD events as outcomes during the lag time were excluded from the analysis. Finally, the operational definition of PD was changed, and the study outcome was re-evaluated according to the following new definitions. First, patients had a diagnostic code for PD accompanied by a PD registration code. Second, patients had a diagnostic code of PD and at least 60 days of prescription records for any PD treatment medications after the first diagnosis. All statistical analyses were performed using SAS statistical software (version 9.4; SAS Institute, Cary, NC, United States). *p < 0.05* was considered significant.

## Results

### Baseline Patient Characteristics

**FIGURE 1:**
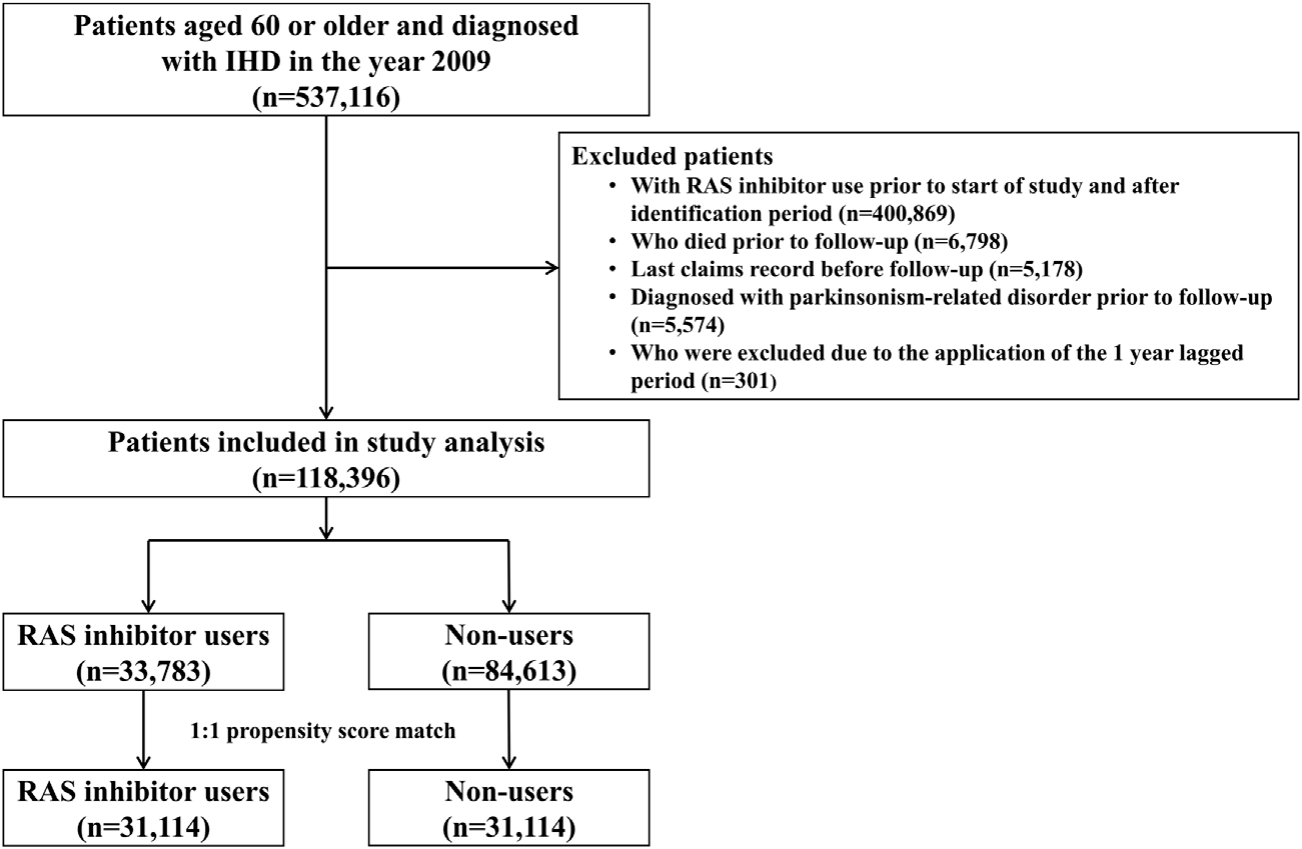
Patient inclusion flowchart. RAS, renin-angiotensin system.

Among the 537,116 patients diagnosed with IHD between January 2009 and June 2009, 31,114 RAS inhibitor users and 31,114 RAS inhibitor non-users were included in the analysis (Figure 1). The baseline characteristics of the study population are shown in Table 1. The mean age (±standard deviation) was 70.3 (±7.1) years, and males accounted for 44.4% of the cohort. The mean duration of follow-up was 7.6 and 7.7 years for RAS inhibitor users and non-users, respectively. After PS matching, the distribution between the two groups became similar for all variables, with standardized differences <0.1. This indicates that the differences between covariates were negligible.

**TABLE 1:** Demographic characteristics of the study population and propensity score matching.

Characteristics	Before matching^a^			After matching^a^		
	Non-users, N (%) (N = 84,613)	RAS inhibitor users, N (%) (N = 33,783)	Standardized difference	Non-users, N (%) (N = 31,114)	RAS inhibitor users, N (%) (N = 31,114)	Standardized difference
Sex			−0.0447			-0.0248
Male	38,661 (45.7)	14,686 (43.5)		14,021 (45.1)	13,638 (43.8)	
Female	45,952 (54.3)	19,097 (56.5)		17,093 (54.9)	17,476 (56.2)	
Age, mean (SD)	69.6 (7.1)	70.3 (7.0)	0.0894	70.4 (7.1)	70.2 (7.7)	-0.0357
Younger than 65 years	23,287 (27.5)	7,894 (23.4)		7,166 (23.0)	7,458 (24.0)	
65 years or older	61,326 (72.5)	25,889 (76.6)		23,948 (77.0)	23,656 (76.0)	
Insurance type			0.121			-0.0068
Health insurance	78,406 (92.7)	30,133 (89.2)		27,996 (90.0)	28,059 (90.2)	
Medical aid	6,207 (7.3)	3,650 (10.8)		3,118 (10.0)	3,055 (9.8)	
Comorbid diseases						
Hypertension	38,580 (45.6)	23,695 (70.1)	0.5132	20,913 (67.2)	21,068 (67.7)	0.0106
Dyslipidemia	35,382 (41.8)	14,667 (43.4)	0.0323	13,243 (42.6)	13,498 (43.4)	0.0166
Diabetes mellitus	22,744 (26.9)	11,403 (33.8)	0.15	9,972 (32.1)	9,923 (31.9)	−0.0034
End-stage renal disease	323 (0.4)	671 (2.0)	0.1487	308 (1.0)	316 (1.0)	0.0026
Stroke	13,808 (16.3)	6,533 (19.3)	0.0789	5,973 (19.2)	5,823 (18.7)	−0.0123
Brain injury	3,465 (4.1)	1,442 (4.3)	0.0087	1,274 (4.1)	1,323 (4.3)	0.0079
Dementia	15,008 (17.7)	7,020 (20.8)	0.0772	6,480 (20.8)	6,271 (20.2)	−0.0166
COPD	8,890 (10.5)	3,440 (10.2)	−0.0106	3,283 (10.6)	3,224 (10.4)	−0.0062
Depression	9,829 (11.6)	4,021 (11.9)	0.0089	3,709 (11.9)	3,689 (11.9)	−0.002
Gout	3,220 (3.8)	1,833 (5.4)	0.0773	1,529 (4.9)	1,535 (4.9)	0.0009
Osteoarthritis	12,144 (14.4)	5,133 (15.2)	0.0237	4,647 (14.9)	4,695 (15.1)	0.0043
Osteoporosis	10,277 (12.2)	3,622 (10.7)	-0.0448	3,378 (10.9)	3,448 (11.1)	0.0072
Severe liver disease	854 (1.0)	322 (1.0)	−.0057	312 (1.0)	298 (1.0)	−0.0046
Concurrent medications						
Antipsychotics	7,573 (9.0)	3,516 (10.4)	0.0493	3,156 (10.1)	3,140 (10.1)	−0.0017
Antiemetics	5,844 (6.9)	2,215 (6.6)	−0.014	2,047 (6.6)	2.078 (6.7)	0.004
Calcium channel antagonists	14,643 (17.3)	3,070 (9.1)	−0.2446	3,060 (9.8)	3,056 (9.8)	−0.0004
Antiepileptics	2,086 (2.5)	1,111 (3.3)	0.0493	956 (3.1)	967 (3.1)	0.002
Trimetazidine	6,130 (7.2)	2,350 (7.0)	−0.0112	2,284 (7.3)	2,185 (7.0)	−0.0123
Mood stabilizer	72 (0.1)	15 (0.1)	−0.016	10 (0)	15 (0.1)	0.008
Beta-blockers	21,748 (25.7)	8,387 (24.8)	−0.0202	8,419 (27.1)	8,143 (26.2)	−0.0201
Dihydropyridine CCBs	22,892 (27.1)	9,370 (27.7)	0.0153	8,891 (28.6)	9,195 (29.6)	0.0215
Statins	34,903 (41.3)	14,497 (42.9)	0.0337	13,163 (42.3)	13,343 (42.9)	0.0117
Follow-up duration (years)	7.7 (2.9)	7.7 (2.9)	−0.011	7.6 (2.9)	7.7 (2.9)	0.0287

CCB, calcium channel blocker; COPD, chronic obstructive pulmonary disease; N, number; RAS, renin-angiotensin system; SD, standard deviation.

a Propensity score matching by age, sex, follow-up duration, insurance type, comorbid diseases, and concurrent medications presented in this table.

### RAS Inhibitor Use and the Risk of PD

A total of 1,086 new PD cases were identified from 477,255 person-years of observation in the main analysis. The overall incidence rate was 2.28 per 1,000 person-years. The median time to censor was 9.4 years (interquartile range, 6.1–9.5 years) in those censored with PD over 10 years of follow-up. The incidence rate of PD was significantly lower in RAS inhibitor users than in non-users (2.04 per 1,000 person-years *vs.* 2.51 per 1,000 person-years, *p < 0.0001*).

RAS inhibitor use was significantly associated with a reduced risk of PD after adjusting for confounders (aHR = 0.75; 95% CI = 0.66–0.85). Male sex and aged ≥65 years were associated with an increased risk of PD. Comorbidities of hypertension, dyslipidemia, diabetes mellitus, stroke, brain injury, dementia, COPD, depression, osteoarthritis, and osteoporosis were significantly associated with an increased risk of PD. Consistent use of medications including antipsychotics, antiemetics, calcium channel antagonists, antiepileptics, trimetazidine, and beta-blockers was significantly associated with an increased risk of PD. Statins were significantly associated with a decreased risk of PD (Table 2).

**TABLE 2:** Cox regression analysis on the association of Parkinson’s disease with renin-angiotensin system inhibitor use and confounding factors (N = 62,228).

	Number of subjects	Person-years	Number of events	Incidence rate^b^	Unadjusted HR (95% Cl)	Adjusted HR (95% Cl)^c^	*p*-value
RAS inhibitor use	31,114	239,923	490	2.04	0.81 (0.72–0.92)	0.75 (0.66–0.85)	<0.0001
Male sex	27,659	206,284	600	2.91	1.06 (0.94–1.20)	1.26 (1.11–1.43)	0.0004
Age							
Younger than 65 years	14,624	128,618	170	1.32	1.00	1.00	
65 years or older	47,604	348,637	916	2.63	1.98 (1.68–2.34)	1.29 (1.08–1.53)	0.0041
Insurance type							
Health insurance	56,055	435,761	965	2.21	1.00	1.00	
Medical aid	6,173	41,494	121	2.92	1.32 (1.09–1.59)	0.90 (0.74–1.09)	0.2601
Comorbid diseases							
Hypertension	41,981	318,066	919	2.89	2.76 (2.34–3.26)	1.49 (1.25–1.77)	<0.0001
Dyslipidemia	26,741	214,493	782	3.65	3.16 (2.77–3.61)	1.94 (1.63–2.30)	<0.0001
Diabetes mellitus	19,895	144,971	624	4.30	3.10 (2.75–3.49)	1.71 (1.51–1.94)	<0.0001
End-stage renal disease	624	3,522	14	3.98	1.74 (1.03–2.96)	1.45 (0.85–2.47)	0.1680
Stroke	11,796	79,295	593	7.48	6.04 (5.36–6.81)	2.11 (1.85–2.41)	<0.0001
Brain injury	2,597	17,704	296	16.72	9.73 (8.51–11.12)	2.49 (2.14–2.90)	<0.0001
Dementia	12,751	87,288	750	8.59	9.98 (8.77–11.35)	4.28 (3.69–4.96)	<0.0001
COPD	6,507	38,996	351	9.00	5.38 (4.74–6.12)	1.92 (1.66–2.21)	<0.0001
Depression	7,398	52,196	602	11.53	10.14 (8.99–11.42)	2.82 (2.46–3.23)	<0.0001
Gout	3,064	24,510	98	4.00	1.84 (1.49–2.26)	1.05 (0.85–1.30)	0.6696
Osteoarthritis	9,342	67,227	569	8.46	6.73 (5.97–7.58)	2.90 (2.53–3.32)	<0.0001
Osteoporosis	6,826	50,737	422	8.32	5.35 (4.74–6.05)	1.93 (1.68–2.22)	<0.0001
Severe liver disease	610	2,692	26	9.66	4.30 (2.91–6.35)	1.45 (0.96–2.18)	0.078
Concurrent medications^a^							
Antipsychotics	6,296	41,559	480	11.55	8.30 (7.36–9.35)	1.93 (1.68–2.22)	<0.0001
Antiemetics	4,125	20,099	404	20.10	13.72 (12.12–15.52)	4.38 (3.78–5.09)	<0.0001
Calcium channel antagonists	6,116	46,886	230	4.91	2.47 (2.14–2.86)	1.42 (1.22–1.65)	<0.0001
Antiepileptics	1,923	11,780	145	12.31	6.06 (5.09–7.22)	1.42 (1.18–1.72)	0.0002
Trimetazidine	4,469	33,339	195	5.85	2.92 (2.50–3.41)	1.47 (1.25–1.74)	<0.0001
Mood stabilizer	25	176	3	17.05	7.58 (2.45–23.50)	1.53 (0.49–4.83)	0.4669
Beta-blockers	16,562	124,145	556	4.48	2.99 (2.65–3.37)	1.73 (1.52–1.97)	<0.0001
Dihydropyridine CCBs	18,086	139,903	434	3.10	1.61 (1.43–1.82)	1.07 (0.94–1.22)	0.3282
Statins	26,506	218,405	688	3.15	2.06 (1.82–2.33)	0.79 (0.68–0.93)	0.0034

CCB, calcium channel blocker; CI, confidence interval; COPD, chronic obstructive pulmonary disease; HR, hazard ratio; RAS, renin-angiotensin system.

a The complete list of concurrent medications is presented in Supplementary Table S3.

b The incidence rate was presented per 1,000 person-years.

c Adjusted for all covariates presentied in this table with a multivariate Cox Proportional Hazard model for Parkinson`s disease.

A significantly reduced risk of PD was observed with the use of ARBs (aHR = 0.74; 95% CI = 0.65–0.85). However, ACEI use was not significantly associated with the risk of PD (aHR = 0.91; 95% CI = 0.77–1.08). The risk of PD was significantly reduced with the use of BBB-crossing RAS inhibitors (aHR = 0.67; 95% CI = 0.57–0.78), but not with the use of non-BBB-crossing RAS inhibitors. Regarding the risk of PD by drug type in relation to the degree of BBB penetrability, only the use of BBB-crossing ARBs showed significant decreases in PD incidence (aHR = 0.62; 95% CI = 0.53–0.74) (Table 3).

**TABLE 3:** Risk of Parkinson’s disease by type of renin-angiotensin system inhibitors and central nervous system penetration (N = 62,228).

	Number of subjects^a^	Person-years	Number of events	Incidence rate^b^	Adjusted HR (95% Cl)^c^
RAS inhibitor non-use	31,114	237,332	596	2.51	1.00 (Reference)
Drug classification					
ACEI use	11,897	88,225	173	1.96	0.91 (0.77–1.08)
ARB use	28,589	223,033	441	1.98	0.74 (0.65–0.85)
BBB penetration					
Non-BBB-crossing	23,805	185,832	363	1.95	0.89 (0.77–1.04)
BBB-crossing	24,526	192,591	341	1.77	0.67 (0.57–0.78)
Drug classification and BBB penetration					
Non-BBB-crossing ACEI	2,900	20,857	40	1.92	1.16 (0.84–1.60)
BBB-crossing ACEI	9,936	74,036	148	2.00	0.93 (0.78–1.12)
Non-BBB-crossing ARB	22,794	179,073	349	1.95	0.90 (0.77–1.05)
BBB-crossing ARB	20,464	164,645	267	1.62	0.62 (0.53–0.74)

ACEI, angiotensin-converting enzyme inhibitor; ARB, angiotensin II receptor type 1 blocker; BBB, blood-brain barrier; cDDD, cumulative defined daily dose; CI, confidence.iInterval; DDD, defined daily dose; HR, hazard ratio.

a The number of subjects in each category of drug type is not mutually exclusive, as the definition of each number of subjects was based on the population who used that drug at least once.

b The incidence rate is presented per 1,000 person-years.

c Adjusted for all covariates presented in Table 2 with a multivariate Cox proportional hazard model for Parkinson’s disease.

**FIGURE 2:**
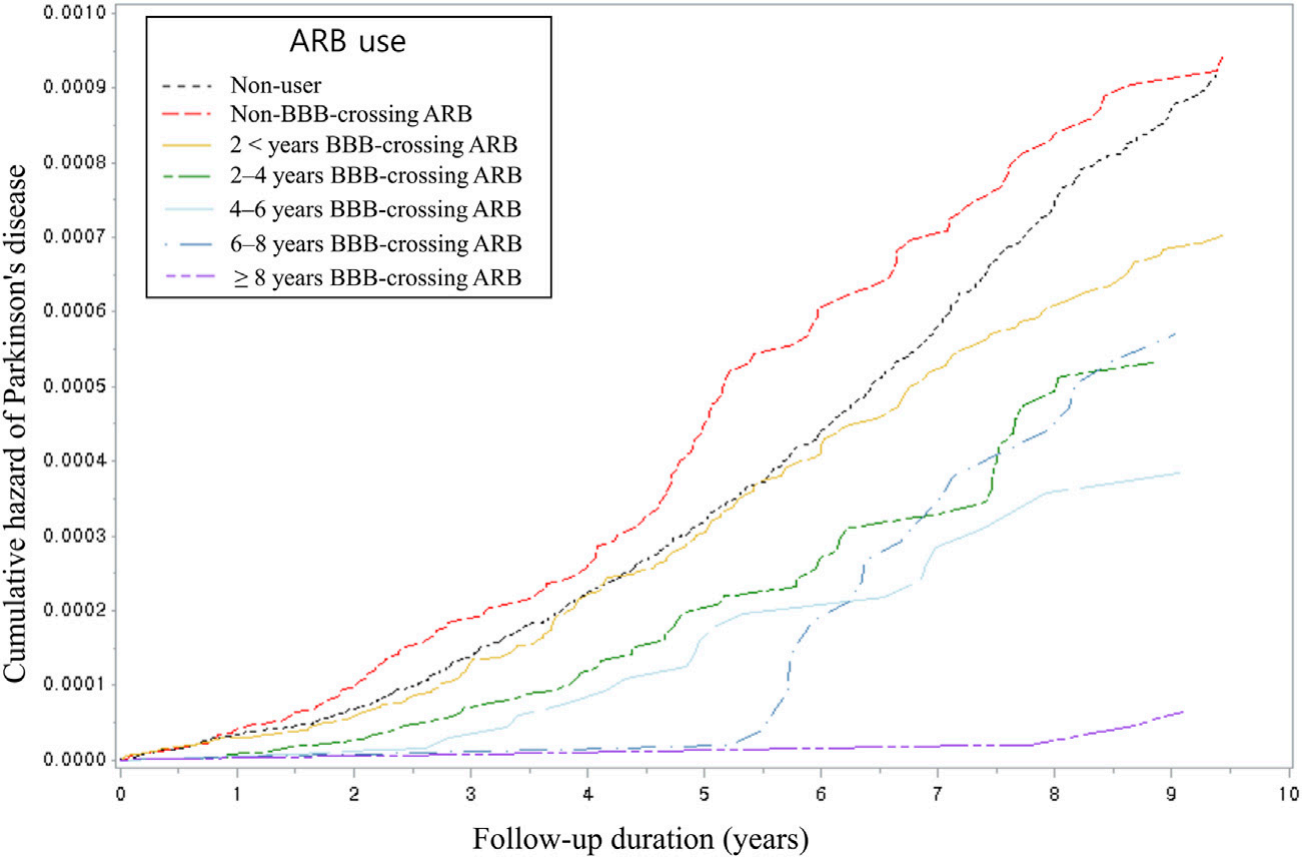
Kaplan-Meier cumulative hazard using Cox proportional hazard analysis by categorized cumulative duration. ARB, angiotensin II type 1 receptor blocker; BBB, blood-brain barrier.

Table 4 presents the PD risk breakdown by cumulative dose, duration, and daily equivalent dosage of BBB-crossing ARBs. In all cDDD groups, the use of BBB-crossing ARBs was significantly associated with a reduced risk of PD. Similar reduced risks of PD were observed in all groups at the daily equivalent dosage. Regarding cumulative duration, RAS inhibitor users who were exposed to ≥2 years of BBB-crossing ARBs showed a lower risk of PD than non-users, whereas those who were exposed to <2 years of BBB-crossing ARBs did not. The risk of developing PD tended to decrease with the increase in the cumulative dose and duration (*P* for trend = 0.03 and <0.01, respectively). Figure 2 shows the Kaplan-Meier curves of the cumulative hazard (1- survival rate) according to the cumulative duration of BBB-crossing ARBs. In the subgroup analyses, the aHRs of BBB-crossing ARBs were comparable in the populations aged<70 years (aHR = 0.65; 95% CI = 0.51–0.83) and ≥70 years (aHR = 0.58; 95% CI = 0.46–0.73). In the sex subgroup analysis, the aHR of BBB-crossing ARBs in the female population (aHR = 0.53; 95% CI = 0.43–0.66) was lower than that in the male population (aHR = 0.75; 95% CI = 0.58–0.96).

**TABLE 4:** Risk of Parkinson’s disease associated with cumulative doses and durations of BBB-crossing ARB use (N = 62,228).

	Number of subjects	Person-years	Number of events	Incidence rate^a^	Adjusted HR (95% Cl)^b^
RAS inhibitor non-use	31,114	237,332	596	2.51	1.00 (Reference)
cDDD of BBB-crossing ARB use					
2 < DDD-years	9,503	69,499	167	2.40	0.80 (0.66–0.97)
2‒4 DDD-years	3,201	25,735	40	1.55	0.48 (0.34–0.67)
4‒6 DDD-years	2,197	18,762	23	1.23	0.60 (0.40–0.92)
6‒8 DDD-years	1,716	15,228	20	1.31	0.60 (0.38–0.95)
≥8 DDD-years	3,847	35,421	17	0.48	0.23 (0.14–0.38)
*P* for trend^c^					0.03
Duration of BBB-crossing ARB use					
2 < years	9,865	71,886	171	2.38	0.82 (0.68–1.00)
2–4 years	3,542	28,621	52	1.82	0.55 (0.41–0.75)
4–6 years	2,553	22,235	20	0.90	0.43 (0.27–0.69)
6–8 years	1,992	18,259	21	1.15	0.55 (0.35–0.86)
≥8 years	2,512	23,644	3	0.13	0.07 (0.02–0.21)
*P* for trend^c^					<0.01
Daily equivalent dosage of BBB-crossing ARB use					
<1 DDD/Day	4,017	32,688	57	1.74	0.65 (0.49–0.86)
1‒2 DDD/Day	14,003	113,235	179	1.58	0.61 (0.51–0.73)
≥2 DDD/Day	2,444	18,722	31	1.66	0.63 (0.44–0.91)
*P* for trend^c^					0.29

ARB, angiotensin II receptor type 1 blocker; BBB, blood-brain barrier; cDDD, cumulative defined daily dose; CI, confidence interval; DDD, defined daily dose; HR, hazard ratio.

a The incidence rate was presented per 1,000 person-years.

b Adjusted for all covariates presented in Table 2, and non-BBB-crossing ARB with a multivariate Cox proportional hazard model for Parkinson’s disease.

c *P* for trend was calculated by using multivariate Cox proportional hazard regression.

### Sensitivity Analyses

Sensitivity analyses for aHRs of RAS inhibitor use for PD are shown in Table 5. The PD risk associated with RAS inhibitor use remained lower than that the risk associated with RAS inhibitor non-use in all sensitivity analyses according to index date shifting, lengthening of the lagged period, and changing the outcome definitions.

**TABLE 5:** Sensitivity analyses of adjusted hazard ratios of renin-angiotensin inhibitor use for Parkinson’s disease.

	Number of subjects	Person-years	Number of events	Incidence rate^a^	Adjusted HR (95% Cl)^b^
Shifting the index date					
July 1. 2009. (main)	31,114	239,923	490	2.04	0.75 (0.66–0.85)
July 1. 2010	43,130	304,837	615	2.02	0.78 (0.70–0.87)
July 1. 2011	48,050	306,441	593	1.94	0.77 (0.69–0.86)
Lengthening lagged period					
1-year lagged period (main)	31,114	239,923	490	2.04	0.75 (0.66–0.85)
3-year lagged period	28,001	176,266	368	2.09	0.81 (0.71–0.94)
5-year lagged period	25,283	121,748	242	1.99	0.76 (0.64–0.90)
Changing the outcome definition					
ICD-10 + drug prescription+ PD registration code (main)	31,114	239,923	490	2.04	0.75 (0.66–0.85)
ICD-10 + PD registration code	31,119	239,864	521	2.17	0.81 (0.72–0.91)
ICD-10 + drug prescription (≥60 days)	31,145	239,907	569	2.37	0.83 (0.74–0.93)

CI, confidence interval; HR, hazard ratio; ICD-10, International Classification of disease 10^th^ Revision; PD, Parkinson’s disease.

a The incidence rate was presented per 1,000 person-years.

b Adjusted for all covariates presented in Table 2 with a multivariate Cox proportional hazard model for Parkinson’s disease.

## Discussion

In this nationwide population-based longitudinal cohort study, IHD patients treated with RAS inhibitors, particularly ARBs, had a lower risk of developing PD than did those who did not use RAS inhibitors. To date, few clinical studies have investigated the effect of RAS inhibitors on PD, and most of them have reported insignificant associations (Becker et al., 2008; Ritz et al., 2010; Warda et al., 2019). Compared to previous studies, our study focused on the effect of RAS inhibitors in the older IHD population. Furthermore, we adopted a robust study design, including analysis of a nationwide cohort with PS matching and landmark analysis, application of the lag-time to avoid reverse causation, adjustment of all possible confounders (Ascherio and Schwarzschild, 2016; Jafari et al., 2013; S. ; Kim et al., 2020; C. H. ; Li et al., 2015; Mullapudi et al., 2016; Rugbjerg et al., 2010; Torsney et al., 2014; Tryc et al., 2013; Vale et al., 2018; Wang et al., 2018), sufficient follow-up, and various sensitivity analyses. Importantly, to our best knowledge, this is the first study to evaluate the effect of BBB-penetration properties of RAS inhibitors on PD occurrence. The results showed that the risk of PD was only significantly reduced in patients who used BBB-crossing ARBs. This association was robust in patients exposed to a larger cumulative dose or longer duration of BBB-crossing ARBs. This result supports that BBB-crossing ARBs should be considered as one of the clinical options for preventing PD in patients with IHD primarily using RAS inhibitors. Moreover, our findings, which were derived from real-world data with 10 years of follow-up and included an evaluation of the impact of cumulative duration and BBB penetration, could add evidence to elucidate the conflicting results reported by previous studies regarding the preventive effect of RAS inhibitors on PD.

Our assessment of the risk of PD according to the type of RAS inhibitors (ACEIs and ARBs) revealed that only ARBs, but not ACEIs, were associated with a lower risk of PD compared with RAS inhibitor non-use. Although, similar to ARBs, the neuroprotective effect of ACEIs was supported by theoretical and experimental evidence, clinical evidence for an association between ACEIs and PD is lacking (Levi Marpillat et al., 2013; N. C. ; Li et al., 2010). Given that ACEIs and ARBs exert RAS inhibition at different sites, the discrepancy in PD risk might be attributable in part to the mechanism of action exerted by each drug class. ACEIs act upstream of the RAS pathway by inhibiting the synthesis of Ang II, which blocks both AT_1_ and AT_2_ receptors. Meanwhile, ARBs bind to the AT_1_ receptor directly to inhibit activation and may relatively increase the activity of endogenous Ang II at the AT_2_ receptor. This counteracts the neurodegenerative effect by AT_1_ activation (Mertens et al., 2010; Gebre et al., 2018). Moreover, blocking the AT_1_ receptor can maintain the elevated Ang-(1-7) by the ACE-sparing effect and indirectly upregulate the conversion of Ang II into Ang-(1-7). The Ang-(1-7)/Mas receptor axis could alleviate the pro-oxidative effects of the Ang II/AT_1_ axis in dopaminergic neurons (Wright et al., 2015; Gironacci et al., 2018). The conversion of excess Ang II to Ang IV also stimulates an increase in dopaminergic neurotransmission in the brain (Braszko, 2004). Additionally, ARBs may exert neuroprotective effects via RAS-independent mechanisms, such as proliferator-activated receptor gamma-associated peroxisome induction inhibition (Garrido-Gil et al., 2012). Further research is warranted to elucidate the specific mechanisms involved in the differences in neuroprotective activity between ACEIs and ARBs.

To the best of our knowledge, this is the first comprehensive study to evaluate PD risk based on the level of BBB penetration of RAS inhibitors. The results of this study indicated that only the use of BBB-crossing ARBs was associated with a reduced risk of PD. Our finding is noteworthy, as the preventive effect of RAS inhibitors on PD was based on a central-acting mechanism. A recent animal study showed that AT_1_ and AT_2_ form AT1/2 heterodimers, which are expressed in both striatal neurons and microglia in the central nervous system (Rivas-Santisteban et al., 2020). Moreover, some experimental studies on the effects of telmisartan and candesartan, which are typical ARBs known to penetrate the BBB, showed an improvement in dopaminergic and mitochondrial functions in an animal PD model (Sekar et al., 2018; Ray et al., 2021). Previous clinical studies on dementia also showed that BBB-crossing RAS inhibitors have better neuroprotective effects than non-BBB-crossing agents (Ho et al., 2021; Ouk et al., 2021). Thus, our real-world evidence suggests the potential value of BBB-crossing ARBs as a prophylactic or disease-modifying therapy for PD. Further studies evaluating the effects of individual ARBs on PD are required based on their BBB penetration ability measured using *in vitro* models and their pharmacodynamic properties such as receptor affinity.

Significantly reduced PD risks were observed in individuals with a greater cumulative dose and longer duration of BBB-crossing ARB use. Similar reduced risks of PD were observed regardless of the daily equivalent dosage. These results indicate that the exposure duration, rather than the dose of BBB-crossing ARBs, might be an influencing factor for this trend. Long-term blockade of the AT_1_ receptor by ARB administration may result in neurological improvement in cerebral ischemia and attenuation of neuronal damage (Culman et al., 2002; Ozacmak et al., 2007). Previous clinical studies have also investigated the duration-response relationship of RAS inhibitors in other neurodegenerative diseases. These studies showed that a longer duration of RAS inhibitor use is associated with a lower risk of neurodegenerative diseases (Davies et al., 2011; Lin et al., 2015). Consistent with these findings, our results identified the long-term effects of RAS inhibitors in the prevention of PD in humans. However, a more robust study to evaluate the long-term effects is required. Interestingly, the protective effect of BBB-crossing ARBs on PD was more remarkable in female than in male. Sex-specific differences in PD risk have been attributed to various potential mechanisms, including hormonal effects and brain RAS expression (Sullivan, 2008; Rodriguez-Perez et al., 2011; Labandeira-Garcia et al., 2016; Sumien et al., 2021). Moreover, enhanced effectiveness of ARBs in females has been shown in both clinical and experimental studies, according to sex differences in the AT_1_/AT_2_ receptor expression ratio (Okumura et al., 2005; Sullivan, 2008). Therefore, our results might be attributed to sex differences in response to brain RAS stimulation and inhibition, as well as to the estrogen-induced dopaminergic neuroprotective effect.

This study had some limitations. First, the study population selection protocol was vulnerable to selection bias. Although the PS matching method may minimize the treatment assignment bias by assembling a sample in which confounders are balanced between RAS users and non-users, some eligible patients, whose characteristics can be related to both the treatment and outcome, were excluded from the study. We also considered the control group as non-users and not active comparators such as those using beta-blockers and calcium channel blockers. Using an active comparator design could reduce selection bias (Yoshida et al., 2015). To address the potential selection bias, we adjusted for various confounders including medications by precise definition. Second, the study findings cannot be generalized to the entire population. The effect of RAS inhibitor use on PD was evaluated in IHD patients, which could affect the incidence of parkinsonism, including PD (Q. Li et al., 2018). Thus, the association between RAS inhibitor use and PD in the general population should be investigated in further studies. Third, our findings could not be extrapolated to young-onset PD occurring in populations aged <50 years because we included a cohort of patients aged ≥60 years. Given that genetics may play a role in young-onset PD (Laperle et al., 2020), additional research on genetic and environmental factors is required. Fourth, as all data used in this study were based on secondary claims data of HIRA, we could not confirm clinical information such as symptoms, laboratory measurements, and the Unified Parkinson’s disease Rating Scale. Given that an accurate diagnosis of PD should be based on clinical and imaging biomarker correlations, the PD cases in our study may have been underestimated or overestimated. Further studies with complete clinical information are warranted to confirm the association of PD progression or motor symptom improvement with RAS inhibitor use (Louis et al., 2009; Kim et al., 2017). However, we used a PD registration code that excludes secondary parkinsonism, which is considered to have a high accuracy for copayment reduction, along with the ICD-10 codes for PD, into the outcome. Additionally, we adopted several approaches to perform sensitivity tests to confirm the robustness and validity of our findings.

In conclusion, our nationwide cohort study highlights that the use of RAS inhibitors, especially BBB-crossing ARBs, was associated with a reduction in the risk of PD in IHD patients. The risk of PD showed a decreasing trend with a higher cumulative duration of BBB-crossing ARBs. These findings could provide insight into the development of disease-modifying drug candidates for PD, adding to existing evidence.

## Data Availability

The data analyzed in this study was obtained from the Health Insurance Review and Assessment Service, the following licenses/restrictions apply: data used in this study are handled and stored by the Health Insurance Review and Assessment Service. Requests to access these datasets should be directed to https://www.hira.or.kr.
