# Supplementary material for: Protective Effect of Renin-Angiotensin System Inhibitors on Parkinson’s Disease: A Nationwide Cohort Study
Source: Front Pharmacol. 2022 Mar 3;13:837890. doi: 10.3389/fphar.2022.837890 (PMC8927987; doi:10.3389/fphar.2022.837890)
Supplement: Supplementary file 1 [file DataSheet1.docx]

Supplementary Material

## Supplementary Figures

**
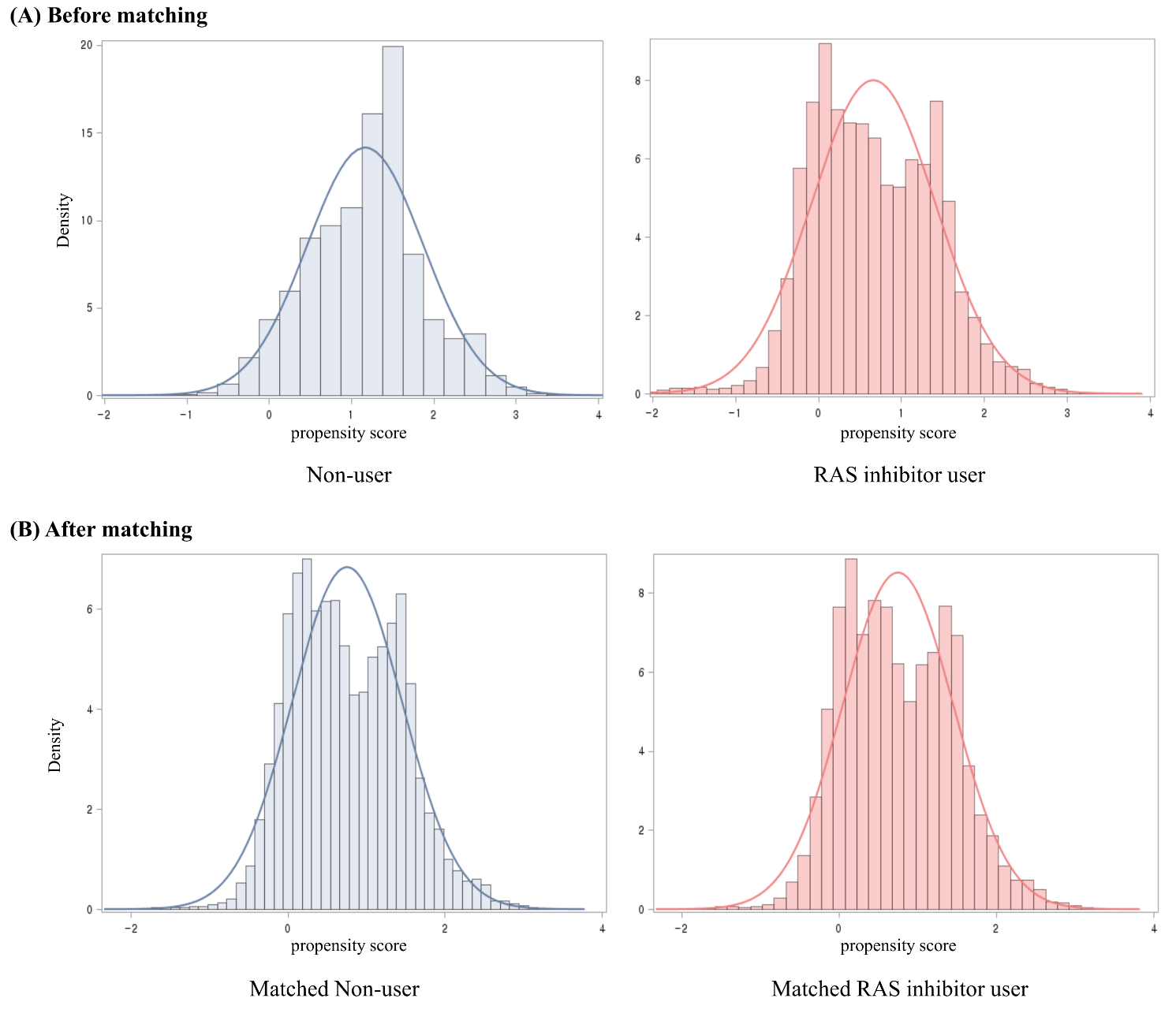
**

**Supplementary Figure 1.** Distribution of propensity score between RAS inhibitor users and non-users before (A) and after (B) matching. RAS, renin-angiotensin system.

**
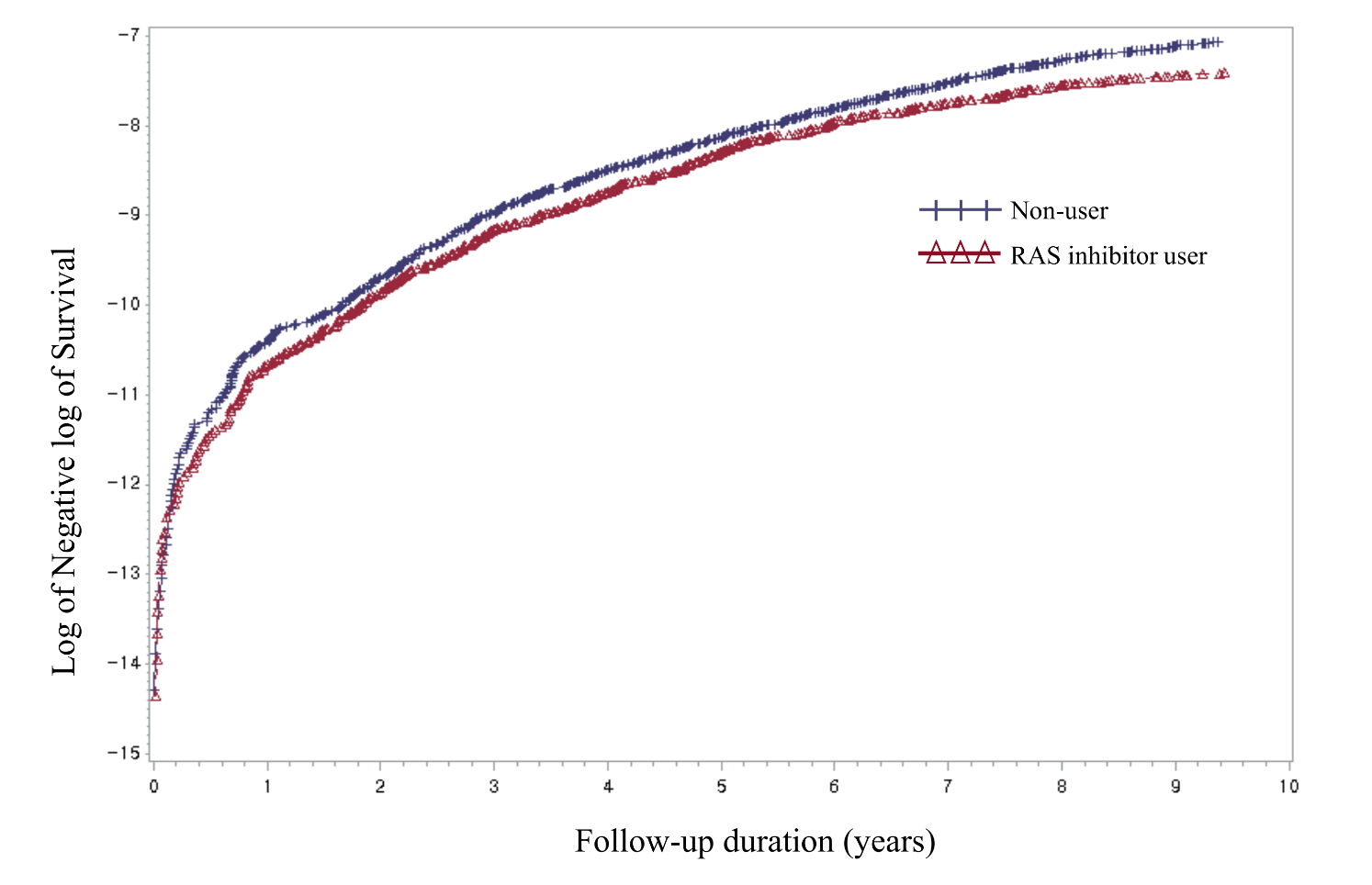
**

**Supplementary Figure 2.** Log (minus log) curves for checking proportional hazard assumption. RAS, renin-angiotensin system.

## Supplementary Tables

**Supplementary Table 1.** List of renin-angiotensin system inhibitors

| **Drug class** | **Drug name** |
| --- | --- |
| **Angiotensin-converting enzyme inhibitors** | Alacepril |
|  | Benazepril |
|  | Captopril^a^ |
|  | Cilazapril |
|  | Delapril^a^ |
|  | Enalapril |
|  | Fosinopril^a^ |
|  | Imidapril |
|  | Lisinopril^a^ |
|  | Moexipril |
|  | Perindopril^a^ |
|  | Quinapril |
|  | Ramipril^a^ |
|  | Temocapril^a^ |
|  | Trandolapril^a^ |
|  | Zofenopril^a^ |
| **Angiotensin II receptor type 1 blockers** | Azilsartan^a^ |
|  | Candesartan^a^ |
|  | Eprosartan |
|  | Fimasartan^a^ |
|  | Irbesartan |
|  | Losartan |
|  | Olmesartan |
|  | Telmisartan^a^ |
|  | Valsartan^a^ |

^a^ Blood-brain barrier penetration

**Supplementary Table 2.** List of ICD-10 codes for comorbid diseases

| **Disease** | **ICD-10 codes** |
| --- | --- |
| Hypertension | I10-I15 |
| Dyslipidemia | E78 |
| Diabetes mellitus | E10-E14 |
| End stage renal disease | N18.5 |
| Stroke | I60-I69, G45-G46 |
| Brain injury | S00-S09 |
| Dementia | G30, F00-F03 |
| Chronic obstructive pulmonary disease | J41-J44 |
| Depression | F32-F33, F34.1, F38.1 |
| Gout | M10 |
| Osteoarthritis | M15-M19 |
| Osteoporosis | M80-M82 |
| Severe liver disease | I85.0, I85.9, I86.4, I98.2, K70.4, K71.1, K72.1, K72.9, K76.5-K76.7 |

ICD, International Classification of Disease 10^th^ Revision.

**Supplementary Table 3.** List of concurrent medications

| **Drug Class** | **Drug name** |
| --- | --- |
| ***Antipsychotics*** | Amisulpiride, Aripiprazole, Bromperidol, Chlorpenthixol, Chlorpromazine, Clozapine, Haloperidol, Levomepromazine, Molindone, Olanzapine, Paliperidone, Perphenazine, Pimozide, Quetiapine, Risperidone, Sulpiride^a^, Thiothixene, Tiapride, Trifluoperazine, Ziprasidone, Zotepine, Zuclopenthixol |
| ***Antiemetics*** | Clebopride |
|  | Levosulpiride |
|  | Metoclopramide |
|  | Sulpiride^a^ |
| ***Calcium channel antagonists*** | Diltiazem |
|  | Flunarizine |
|  | Verapamil |
| ***Antiepileptics*** | Levetiracetam |
|  | Phenytoin |
|  | Valproate |
| ***Dopamine depleter*** | Tetrabenazine |
| ***Mood stabilizer*** | Lithium |
| ***Beta-blockers*** | Atenolol, Betaxolol, Bevantolol, Bisoprolol, Carteolol, Carvedilol, Celiprolol, Esmolol, Labetalol, Metoprolol, Nadolol, Nebivolol, Propranolol, Sotalol |
| ***Calcium channel blockers (Dihydropyridine)*** | Amlodipine, Barnidipine, Benidipine, Cilnidipine, Efonidipine, Felodipine, Isradipine, Lacidipine, Lercanidipine, Manidipine, Nicardipine, Nifedipine, Nilvadipine, Nimodipine, Nisoldipine, Nitrendipine |
| ***Statins*** | Atorvastatin, Fluvastatin, Lovastatin, Pitavastatin, Pravastatin, Rosuvastatin, Simvastatin |

^a^Some products of sulpiride were approved as antiemetics by Ministry of Food and Drug Safety.
